# Properties of Peanut (KAC431) Protein Hydrolysates and Their Impact on the Quality of Gluten-Free Rice Bread

**DOI:** 10.3390/foods9070942

**Published:** 2020-07-16

**Authors:** Suphat Phongthai, Nuttapon Singsaeng, Rossarin Nhoo-ied, Thipubol Suwannatrai, Regine Schönlechner, Kridsada Unban, Warinporn Klunklin, Thunnop Laokuldilok, Yuthana Phimolsiripol, Saroat Rawdkuen

**Affiliations:** 1 School of Agro-Industry, Faculty of Agro-Industry, Chiang Mai University, Chiang Mai 50100, Thailand; ntp026@gmail.com (N.S.); rossarin.fern@gmail.com (R.N.-i.); tipubol2540@gmail.com (T.S.); karn039@gmail.com (K.U.); wklunklin@gmail.com (W.K.); thunnop.l@cmu.ac.th (T.L.); yuthana.p@cmu.ac.th (Y.P.); 2 Cluster of High Value Product from Thai Rice for Health, Chiang Mai University, Chiang Mai 50100, Thailand; 3 Institute of Food Technology, Department of Food Science and Technology, BOKU-University of Natural Resources and Life Sciences, 18, 1190 Vienna, Austria; regine.schoenlechner@boku.ac.at; 4 Unit of Innovative Food Packaging and Biomaterials, School of Agro-Industry, Mae Fah Luang University, Chiang Rai 57100, Thailand; saroat@mfu.ac.th

**Keywords:** peanut, protein hydrolysate, degree of hydrolysis, rice, gluten-free bread

## Abstract

Protein hydrolysates (PH) with a degree of hydrolysis (DH) of 5%, 10%, and 13% from two varieties of peanut were prepared using two commercial enzymes, Alcalase and Flavourzyme. The content of essential amino acids (30,290 mg/100 g) and hydrophobic amino acids (34,067 mg/100 g) of the peanut variety Kalasin 2 (KAC431) protein was higher than that of a common variety, Kalasin 1 (KAC1) (*p* < 0.05). The protein molecular weight distributions of the two varieties of peanut detected by sodium dodecyl sulfate polyacrylamide gel electrophoresis (SDS-PAGE) were similar, ranging from 15 to 75 kDa, with a major protein band at 50–75 kDa. The antioxidant and functional properties of derived PHs were influenced by DH. Although the foaming ability of protein was improved by DH5%, it was obviously decreased upon increasing DH further. The best emulsifying properties were observed in PH with DH5% (*p* < 0.05). The incorporation of PH with a small DH, especially when produced using Flavourzyme, had a highly positive impact on the specific volume and relative elasticity of gluten-free bread. The effect of PHs on bread quality was highly correlated with their functional properties. This study suggests that partially enzymatically modified proteins are suitable for incorporation in food products such as bread and other gluten-free products.

## 1. Introduction

Generally, wheat flour is used as the main ingredient in most bread because it contains the storage protein gluten, a critical factor for dough structure. However, gluten has to be eliminated from the diet for celiac consumers since it causes some adverse effects in the small intestine that can lead to malabsorption and cause diarrhea, indigestion, bloating, and weight loss [1]. Currently, the development of gluten-free bread has gained much attention from researchers and/or food recipe developers worldwide due to its highest popularity among gluten-free baked products. Many hydrocolloids, such as egg white solids [2], inulin [3], and gums [4], have been studied for substituting or imitating the gluten network in gluten-free breads. Apparently, the gluten-free breads that are now commercially available in current markets, which are made from gluten-free flours or starches, have yet to receive acceptability from consumers due to their poor appearance and low nutrition values.

Protein, one of the most popular hydrocolloids, usually exerts several functional properties depending on its three-dimensional structures. It is widely applied to gluten-free products with the goal of enhancing quality and nutrition value. Based on our previous study [5], the addition of 2% rice bran protein concentrate is optimal for improving the quality of gluten-free bread, whereas the addition of more than 2% protein has a negative effect on gluten-free bread quality. This is due to the restricted functional properties of the native protein, leading to poor bread quality. However, in some reported studies it was discovered that the functional properties of protein can be effectively modified and extended by enzymatic hydrolysis [6,7,8], resulting in a wider range of applications. Therefore, the use of protein hydrolysates to improve bread quality may be useful for the development of high-quality gluten-free bread.

Peanut (*Arachis hypogaea*) has long been consumed as a human food because it is rich in oil and vitamins, as well as protein. Many varieties of peanut have been commonly cultivated in Thailand, such as Kalasin 1 (KAC1), Thainan 9, Konkaen 4, etc. Recently, peanut cv. Kalasin 2 (KAC431), which is suitably cultivated in Mae Hong Son Province, Thailand, has achieved considerable popularity over other varieties due to its outstanding taste. As the production has been increasing each season, it is of interest and a challenge to investigate alternative ways to use KAC431 as a food ingredient, rather than direct consumption.

In this study, KAC431 and a common peanut (cv. KAC1) were used as sources of protein. Protein hydrolysates prepared using two commercial enzymes with different degrees of hydrolysis were assessed for their chemical and functional properties, and further applied in gluten-free rice bread. The impact of selected protein hydrolysates on the bread quality parameters, such as weight loss, specific volume, crumb firmness, and relative elasticity, of the final products was investigated.

## 2. Materials and Methods

### 2.1. Materials

Peanuts (KAC1 and KAC431) were purchased from a local market in Mae Hong Son Province, Thailand. Alcalase (2.99 Units/mL) was purchased from EMD Chemicals, Inc. (San Diego, CA, USA). Flavourzyme (20,000 Units/g) was provided by Assistant Professor Dr. Akkasit Jongjareonrak. Rice flour was purchased from the Bangkok Inter Food Co., Ltd. (Bangkok, Thailand).

### 2.2. Methods

#### 2.2.1. Extraction of Peanut Proteins

Peanuts were de-shelled and dried in a hot-air oven at 70 °C for 16 h. Dry peanuts were ground, defatted using hexane (1:5) for 6 h, and dried in a hot-air oven at 60 °C for 16 h. The defatted peanut powder was mixed with distilled water (1:5) and the pH of slurry was adjusted to 10 using 1 M NaOH. The extract was processed using a magnetic stirrer at 50 °C for 1.5 h. The mixture was centrifuged (Super T-21, Kendo Laboratory Products, San Francisco, CA, USA) at 6000 rpm for 10 min and the supernatant was collected. The pH of the solution was adjusted to 4.5 using 1 M HCl and centrifuged again. The protein precipitates were collected and the pH was adjusted to 7.0 before drying using a freeze dryer. The protein yield was calculated by dividing the derived protein powder by the peanut meal used, then multiplying by 100. The extraction was performed in duplicate.

#### 2.2.2. Analysis of Proximate Composition

Protein content was analyzed by the combustion method (Leco FP-528, 601500, Leco Corporation, St. Joseph, MI, USA) with a nitrogen conversion factor of 5.46. Moisture, oil, fiber, ash, and carbohydrate contents were quantified according to the method of the Association of Official Analytical Chemists (AOAC) [9].

#### 2.2.3. Analysis of Amino Acid Profiles

To determine the amino acid composition, peanut proteins were separated and analyzed on a Zebron ZB-AAA GC column (10 mm × 0.25 mm, 0.25 μm film thickness) and detected by GC–MS (6890N; Agilent Technologies, Santa Clara, CA, USA) according to the method of the AOAC [9].

#### 2.2.4. Production of Protein Hydrolysates (PHs)

Hydrolysis of peanut protein was conducted using the pH-stat assay following the method described in a previous study [6]. Flavourzyme (23,400 Units, pH 7.0, 50 °C) and Alcalase (0.81 Units, pH 8.5, 55 °C) were used to prepare protein hydrolysates (PHs) with degrees of hydrolysis (DHs) of 5%, 10%, and 13%, obtaining FDH5%, FDH10%, FDH13%, ADH5%, ADH10%, and ADH13%, respectively. The DH was calculated from the following equation:

DH (%) = (BN_b_/αh_tot_M_p_) × 100 (1)

where B is the volume of NaOH used (mL), N_b_ is the normality of the NaOH, M_p_ is the mass of protein in grams (N × 4.60), h_tot_ is the total number of peptide bonds in the protein substrate, and α is the average degree of dissociation of the α-NH_2_ groups, which can be calculated from the following equation:

α = (10^pH-pKa^/1 + 10^pH-pKa^) (2)

where pH is the optimal pH for the hydrolysis of the enzyme, and pKa was calculated from the parameters of the conditions used.

#### 2.2.5. Chemical and Physical Properties

##### Protein Patterns

The protein patterns of peanut variety KAC431 and KAC1 were investigated using sodium dodecyl sulfate polyacrylamide gel electrophoresis (SDS-PAGE) as described by Laemmli [10]. The protein solutions were mixed with a sample buffer (0.125 M Tris-HCl, pH 6.8, 4% SDS, and 20% glycerol) at a ratio of 1:1 (*v*/*v*). Protein markers and samples (15 µL) were loaded onto 12% of the separation gels and 4% of the stacking gels. The electrophoresis system was set and run under a constant current of 15 mA/gel. The sample-loaded gels were stained overnight in a solution of Coomassie Brilliant Blue R-250 and de-stained in acetic acid/methanol solution under gentle shaking. The molecular weight of proteins in unknown samples was estimated by comparing their electrophoretic mobility with that of known protein standards.

##### Antioxidant Properties

The determination of DPPH (2,2-diphenyl-1-picryl-hydrazyl-hydrate) and ABTS (2,2′-azino-bis-3-ethylbenzthiazoline-6-sulphonic acid) radical scavenging activity was conducted following the method of Phongthai et al. [11] with some modifications. The protein solution (1 mg/mL, 0.5 mL) was mixed with 0.1 mM DPPH (2 mL) thoroughly. The mixture was incubated in the dark for 30 min. The absorbance was read at 517 nm (Genesys™ 10S, Thermo Scientific, Waltham, MA, USA). The values were calculated and reported as the percentage of inhibition activity using the following equation:

Inhibition activity (%) = [(Abs_control_ − Abs_sample_)/Abs_control_] × 100 (3)

where Abs_control_ and Abs_sample_ are the absorbance of the control and sample, respectively.

For ABTS radical scavenging ability, a stock solution, prepared by mixing of 2.6 mM potassium persulfate and 7.4 mM ABTS (1:1), was incubated for 12 h. The stock solution was diluted with DI water (1:60) for use as a working solution. The protein solution (150 µL) was mixed with 28.5 mL of working solution. The mixtures were incubated for 2 h in the dark. The absorbance was measured at 734 nm. The values were calculated and reported as the percentage of inhibition activity.

##### Foaming and Emulsifying Properties

Functional properties, including foaming and emulsifying properties, were determined according to our published study [11]. Protein solutions (1% *w*/*v*, 20 mL) were transferred to a 100 mL cylinder and then homogenized at a speed of 10,000 rpm for 1 min. The initial total volume at 0 min and final volume after 30 min whipping were measured. The foaming activity and stability were calculated from the following equations:

Foaming activity = [(A − B)/B] × 100(4)

Foam stability = (A_30min_ − B)/(A_0min_ − B) × 100 (5)

where A is the volume after whipping (mL) and B is the volume before whipping (mL).

To determine the emulsifying properties, protein solutions (5% *w*/*v*, 10 mL) were mixed with an equal quantity of refined peanut oil and homogenized at 10,000 rpm for 1 min and then centrifuged at 1500× *g* for 5 min. The emulsifying properties were calculated from the following equations:

Emulsifying activity = (A/B) × 100(6)

Emulsifying stability = (A_incubate_/B) × 100 (7)

where A is the volume of the emulsifying layer (mL) after centrifugation, B is the total volume (mL), and A_incubate_ is the emulsifying volume after incubation at 80 °C for 10 min and centrifugation.

#### 2.2.6. Gluten-Free Bread Preparation

The basic bread recipe, containing 100 g rice flour, 1 g Hydroxypropyl methylcellulose (HPMC), 1 g emulsifier (BACOM A100, Bakels, Silverwater, NSW, Australia), 1 g butter, 2 g salt, 3 g instant yeast, and 105 g water, was based on a previous study [5] with some modifications. Peanut protein concentrate, selected PHs, and egg albumen were individually added at 2% (based on flour). Bread produced without the addition of any protein was used as the control sample.

All dry ingredients (HPMC, rice flour, and salt) were mixed together in a mixer (5K5SS, Kitchen Aid^®^, Benton Harbor, MI, USA) for 1 min (speed 2). Afterwards, butter, emulsifier, dry instant yeast, and water were subsequently slowly added. The mixing was continued for 6 min at speed 2. The mixture was incubated at 35 ± 2 °C, 85% ± 3% RH for 30 min in an incubator (LEV141XV, Smeg S.p.A, Guastalla, Italy). The derived batter was divided into two portions, and then proofed at 35 ± 2 °C, 85% ± 3% RH for another 30 min. Baking was conducted at 180 °C for 40 min (ELE-1450A, Heng Wei, Wuxi, JS, China). Baking trials were performed in duplicate.

#### 2.2.7. Bread Quality Determinations

##### Specific Volume and Weight Loss

The specific volume was determined by the rapeseed displacement method as described in the American Association for Clinical Chemistry (AACC) Approved Method 55–50 [12]. The weight of batter and bread was used to calculate the percentage weight loss. All measurements were done in duplicate.

##### Crumb Firmness and Relative Elasticity

The crumb firmness and relative elasticity of breads were determined using a texture analyzer (TA-XT-Plus, 10207, Stable Micro System™ Co., Godalming, Surrey, UK). A 25-kg load cell with a compression probe (P50) was used. A sliced bread cube with dimensions of 4 × 3 × 3 cm (*L* × *W* × *H*) was subjected to a uniaxial compression test (30% compression) with a holding time of 120 s. Crumb firmness represented the maximum force (*F_max_*) of compression. The relative elasticity was calculated by dividing the residual force (*F_res_*) at 120 s by the maximum force, and then multiplying by 100.

#### 2.2.8. Statistical Analysis

Statistical analysis of the experimental data was performed using analysis of variance (ANOVA), and Duncan’s multiple range test (DMRT) was used to compare the means at 95% confidence level (SPSS Version 17.0).

## 3. Results and Discussion

### 3.1. Proximate Composition and Protein Extraction Yield

The proximate composition of defatted peanuts is shown in Table 1. The largest fraction in both peanut meals was protein content, ranging from 35.16% to 42.39%. Protein remaining in KAC431 was significantly higher than in KAC1, by about 17% (*p* < 0.05). Carbohydrate was the second largest fraction, found in the range of 27.03% to 29.42%. The residual oil and crude fiber in KAC1 (18.77% and 10.83%, respectively) were significantly different to those in KAC431 (15.01% and 5.31%, respectively) (*p* < 0.05). Oil content can be reduced by the re-extraction process, increased contact time, or adjustment of the solid–liquid ratio. Furthermore, the ash and moisture content of both peanut meals was less than 5%. As protein was a major component in defatted peanut meal, it could be a potential material for plant-based protein production.

The protein extraction yield obtained was consistent with the protein content in raw material. The extraction yield of KAC431 (43.26%) was 1.16-fold higher than that of KAC1 (37.25%) (*p* < 0.05). However, it was clearly observed that more than 50% protein remained in peanut meal residues; therefore, it might be necessary to carry out a re-extraction process in order to increase extraction yield. These proteins are suitable for use as raw material for the production of PHs as the derived protein concentrates had sufficiently high protein content (74.14% to 77.76%). Likewise, Torruco-Uco et al. [13] successfully prepared PHs with an antihypertensive effect using lima bean (*Phaseolus lunatus*) and jamapa bean (*Phaseolus vulgaris*) protein concentrates, consisting of 63.8–71.8% protein content, as starting materials.

### 3.2. Amino Acid Profiles

The amino acid composition of peanut proteins is listed in Table 2. The amino acid profiles of proteins from the two peanuts were slightly different. Total amino acid in KAC431 (82,223 mg/100 g) was higher than in KAC1 (81,636 mg/100 g). The most prevalent amino acids were glutamic acid (21,108–213,310 mg/100 g), aspartic acid (8336–9267 mg/100 g), and leucine (7130–7885 mg/100 g). The essential amino acid (histidine, isoleucine, leucine, lysine, methionine, phenylalanine, threonine, tryptophan, and valine) content of KAC431 was only 3% higher than that of KAC1. The consumption of about 1.29 g/kg per day of these proteins could meet the amino acid requirement of adults as recommended by a Joint WHO/FAO/UNU Expert Consultation [14].

Moreover, electrically charged and hydrophobic amino acids were found to be the major group in both KAC431 and KAC1 proteins. These types of amino acids are recognized as a critical factor for protein functionality. Electrically charged amino acids play a major role in protein solubility, whereas hydrophobic amino acids have previously been reported to have a strong correlation with foaming and emulsifying properties, as well as some bioactivity such as antioxidant and antihypertension effects [15,16]. Thus, peanut protein concentrates can be used as a starting material for the preparation of a new ingredient that might have good functional properties and bio-functions.

### 3.3. DH and PH Yields

**Figure 1:**
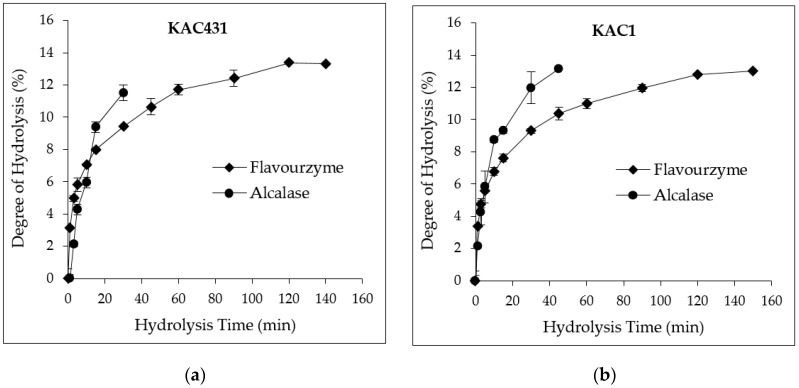
Hydrolysis patterns of peanut proteins (**a**) Kalasin 2 (KAC431) and (**b**) Kalasin 1 (KAC1) by Alcalase (•) and Flavourzyme (♦). Number of replicates: *n* = 2.

Peanut PHs with different DHs were prepared using Flavourzyme and Alcalase. It was found that the efficiency of Flavourzyme to hydrolyze proteins from KAC431 and KAC1 was quite low, even though many units of enzyme were used (Figure 1).

The proteins were rapidly hydrolyzed by Flavourzyme within only the first 20 min. The PHs from KAC431 and KAC1 with DH5% and DH10% were comparatively obtained at 3 and 45 min, respectively, with PH yields ranging from 65.71% to 68.95% (data not shown). After that, protein hydrolysis reached a maximum point with DH13% at 120 min for KAC431 and 150 min for KAC1, then the hydrolysis rate become constant, achieving PH yields of 68.21% to 73.15%. Therefore, the highest DH of PHs in this study was set at 13% to avoid too much enzyme usage, which may cause some interference during the determination of properties. Alcalase showed a greater hydrolysis efficiency although fewer units of enzyme were used. Hydrolysis of proteins from KAC431 and KAC1 was rapidly reached DH5% at 4 min. The PH yield ranged from 63.02% to 71.44%. However, PHs obtained with DH10% and DH13% were derived at 11.5 and 25.5 min for KAC431, and at 18.9 and 45 min for KAC1, respectively. It can be concluded that the protein in KAC431 is more susceptible to either Flavourzyme or Alcalase than KAC1 protein. The protein hydrolysis patterns for Alcalase and Flavourzyme in this study are similar to those for other legumes reported by Torruco-Uco et al. [13]. In contrast, higher proteolytic activity has been reported for Flavourzyme than Alcalase towards the barley protein hordein [17]. The different protein hydrolysis rates might be mainly due to the difference in amino acid sequences/compositions that are specific to the activity of each enzyme.

According to their superior amino acid composition and faster hydrolysis rate, proteins from KAC431 were chosen for the determination of chemical and functional properties, in order to use them as quality improvers for gluten-free rice bread.

### 3.4. Properties of PHs

#### 3.4.1. Protein Patterns

**Figure 2:**
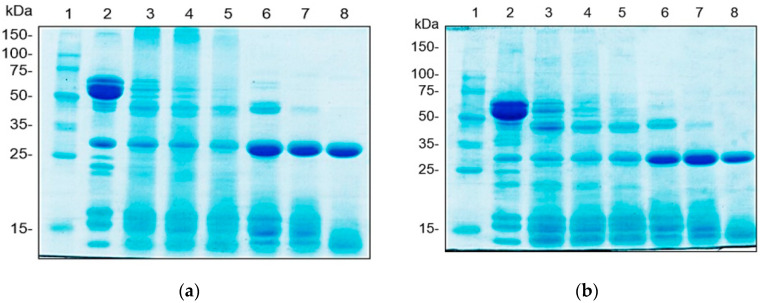
Protein patterns of peanut proteins (**a**) KAC431 and (**b**) KAC1, Lane 1: marker, Lane 2: protein concentrate, Lane 3: ADH5%, Lane 4: ADH10%, Lane 5: ADH13%, Lane 6: FDH5%, Lane 7: FDH10%, Lane 8: FDH13%.

Proteins were separated by SDS-PAGE according to their molecular weight (MW) distribution (Figure 2). The major bands of native peanut proteins were found at 50–75 kDa. This might be a conarachin II, a peanut storage protein, which has MW of 61.0 kDa [18]. The second major band was identified at 28.6 kDa. An additional protein band of KAC431 (Figure 2a) was observed at 23.8 kDa. The number of protein bands was obviously reduced and then disappeared as the DH increased from 5% to 13%. Under Alcalase hydrolysis, proteins with MW of 28.6 and 45.2 kDa remained at every DH, whereas the protein with an MW of 45.2 kDa was completely hydrolyzed by Flavourzyme. The intensity of protein bands, especially at 28.63 kDa, was different among PHs obtained using Alcalase and Flavourzyme. As a band’s intensity can be roughly used to estimate the quantity of protein, it can be concluded that PHs obtained using Flavourzyme may contain more protein with an MW of 28.6 kDa than those obtained using Alcalase.

The difference in the protein pattern was attributed to the function of the enzyme used and the hydrolysis time. Alcalase is an endopeptidase that quickly cleaves internal peptide bonds inside protein molecules with broad specificity, thus creating small peptide fragments, whereas Flavourzyme is a mixture of endo- and exopeptidases that may hydrolyze peptide bonds at a slower rate at the C-terminal and N-terminal ends of protein chains, possibly producing bigger peptides. This result was consistent with the hydrolysis patterns mentioned in Section 3.3.

#### 3.4.2. Antioxidant Properties

**Figure 3:**
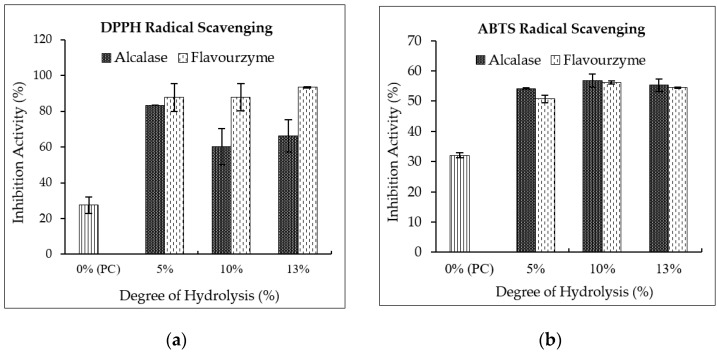
Antioxidant activities (**a**); DPPH radical scavenging, and (**b**); ABTS radical scavenging) of peanut protein concentrate and hydrolysates. Number of replicates: *n* = 3.

The DPPH and ABTS radical scavenging ability of peanut (KAC431) PHs is illustrated in Figure 3a,b. It is clearly seen that enzymatic hydrolysis by Alcalase and Flavourzyme significantly enhanced the radical scavenging ability of peanut proteins, about 2.19–3.40-fold for the DPPH radical, and about 1.58–1.77-fold for the ABTS radical, when compared to non-hydrolyzed proteins (*p* < 0.05). At DH5%, PHs obtained using both enzymes comparatively inhibited the DPPH radical; however, the inhibition activity became very different at DH10% and DH13%. PHs obtained using Flavourzyme showed a higher activity than Alcalase hydrolysate (*p* < 0.05). This might be due to Flavourzyme, a mixture of proteases with both exo- and endopeptidase activity, being more efficient in creating surface hydrophobicity than Alcalase, as also found in cowpea PH [19], allowing a greater dispersion in methanol which was used as a solvent, and thus reacting better with DPPH radicals.

Furthermore, the DH and type of enzyme seemed not to affect the ABTS radical scavenging activity of PHs much. It was clearly seen that PHs with DH10% and DH13% hydrolyzed by either Alcalase or Flavourzyme exhibited similar inhibition activity, ranging from 50.76% to 56.79%. However, ADH5% seemed to be more active in scavenging ABTS radicals than Flavourzyme hydrolysate (*p* < 0.05). As distilled water was used as a solvent in the ABTS system, the PHs derived from Flavourzyme hydrolysis, which were more hydrophobic, possessed a lower ABTS radical scavenging activity than those obtained using Alcalase. A similar observation was previously reported in the study of Vaštag et al. [20]; Alcalase hydrolysates of pumpkin seed protein exhibited about two- to threefold higher ABTS radical scavenging than Flavourzyme hydrolysates.

#### 3.4.3. Functional Properties

**Figure 4:**
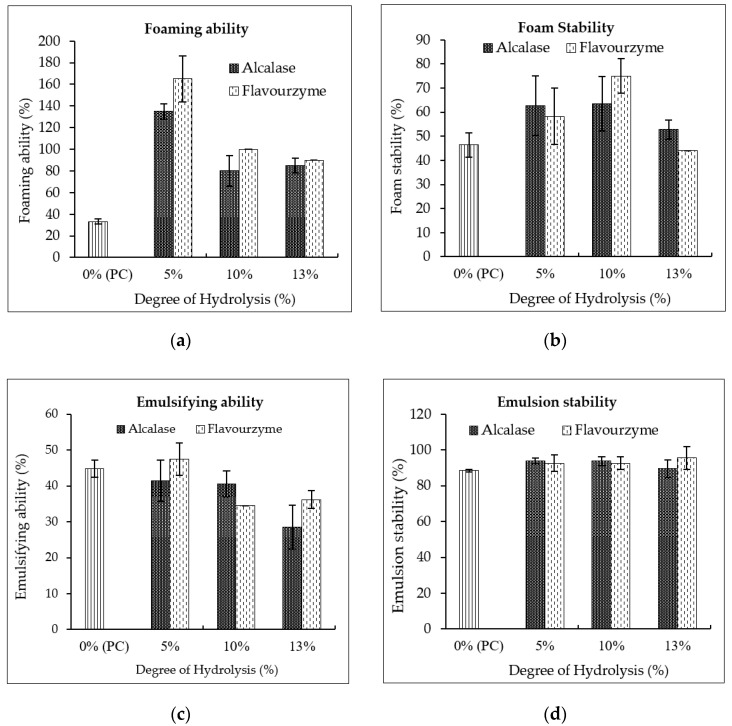
Functional properties (**a**); foaming ability, (**b**); foaming stability, (**c**); emulsifying ability, (**d**); emulsion stability of peanut protein concentrate and hydrolysates. Number of replicates: *n* = 3.

Foaming ability and foam stability are shown in Figure 4a,b. It was found that the foaming ability of protein can be significantly improved by enzymatic modification (*p* < 0.05). Partial hydrolysis by Alcalase and Flavourzyme with DH5% provided outstanding foaming ability, of about 135–165% (*p* > 0.05). This was due to enzymatic hydrolysis resulting in the unfolding of the protein structure, exposing hydrophobic side chains and then improving the hydrophobic–hydrophilic balance [21,22]. These proteins can form a flexible film to envelop air bubbles in their structures. PHs obtained using Flavourzyme had a better foaming ability than those obtained using Alcalase (*p* < 0.05), caused by the function of the enzyme to create a more hydrophobic surface. Bamdad et al. [17] and Segura-Campos et al. [19] also found that Flavourzyme resulted in the better unfolding of the hydrophobic surface of barley and cowpea protein molecules than Alcalase, enhancing interactions at the air–water interface. However, a higher DH induced the greater creation of smaller peptides, and thus the foaming ability decreased by 20.74–39.39% and 22.04–35.45% at DH10% and DH13%, respectively. PHs with DH5% and DH10% had better foam stability than the native protein (DH0%), whereas excessive hydrolysis resulted in the poor stability of foam, as seen for PHs with DH13%. Smaller peptides have less ability to stabilize foam, whereas larger peptides effectively form flexible films around the air bubbles, providing greater foam stability.

Emulsifying ability and emulsion stability are presented in Figure 4c,d. It was observed that a small DH (DH5%) did not change the ability of PHs to emulsify two immiscible liquids of oil and water, resulting in comparable emulsifying abilities (41.38% to 47.41%). Interestingly, the result revealed that a higher DH reduced emulsifying ability (28.45% to 36.20%). Although small peptides can migrate quicker and adsorb at the oil–water interface, they have poor efficiency to reduce interfacial tension, as short-chain peptides may unfold and reorient at the interface to a lesser degree than large peptides [23]. The studies of Phongthai et al. [6] and Jamdar et al. [24] suggested that hydrolysis of peanut and rice bran proteins with a DH of more than 10% can reduce the emulsifying activity of proteins. However, the different DH and enzyme types used in this study had less effect on improving emulsion stability.

According to greater functional properties, especially foaming ability, PHs with DH5% and DH10% were selected to investigate their possible effects on gluten-free bread quality.

### 3.5. Effect of PHs on Gluten-Free Bread Qualities

#### 3.5.1. Weight loss and Specific Volume

Water evaporation during baking accounts for weight loss in baked products. The weight losses of gluten-free rice breads are shown in Table 3. In this study, the lowest weight loss was derived from the control recipe (12.56%, *p* < 0.05). The addition of 2% peanut protein concentrate (KAC431) and 2% egg albumen caused a loss of moisture in gluten-free breads of about 12.57–12.72%. Moreover, upon the incorporation of 0.5–3.5% bovine plasma, >90% of moisture was retained in rice flour-based bread [25]. However, breads incorporating PHs had a significantly higher level of weight loss of about 13.34–13.59% (*p* < 0.05). This is probably due to the diminished water-holding capacity of proteins subjected to enzymatic hydrolysis (data not shown). The smaller protein molecules produced may lose the capacity to hold water in their own structure, resulting in greater moisture loss.

**Figure 5:**
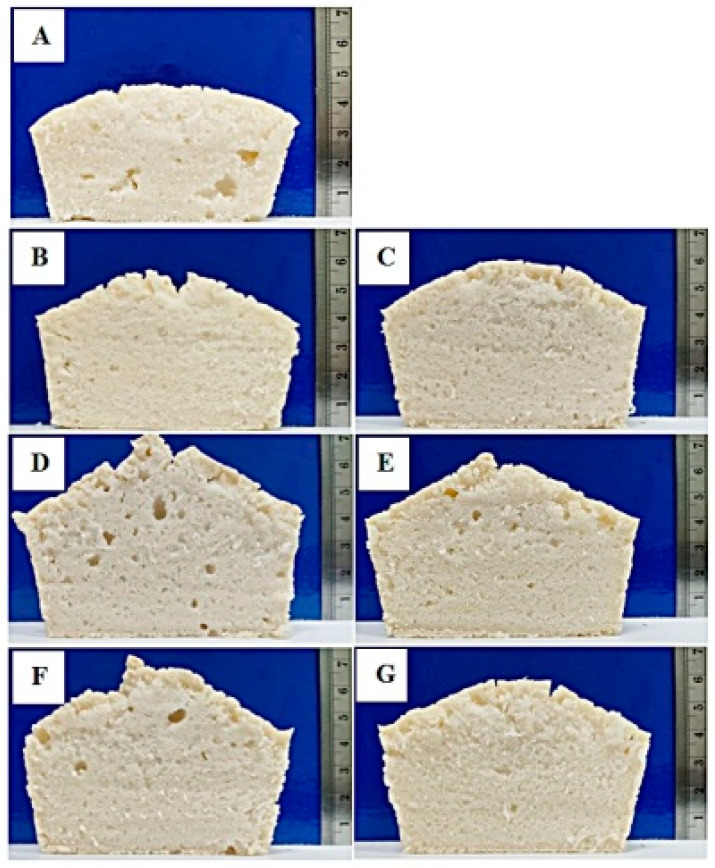
Crumb structure of gluten-free rice breads (**A**); control, (**B**); egg albumen, (**C**); peanut protein concentrate, (**D**); FDH5%, (**E**); FDH10%, (**F**); ADH5%, and (**G**); ADH10%.

The specific volume of gluten-free breads is summarized in Table 3. It was clearly seen that bread without protein added had the lowest specific volume (*p* < 0.05) as also observed in Figure 5a. This is because it is lacking the protein network that provides elastic and cohesive properties, so gas retention of batter is mainly controlled by viscosity, leading to poor specific loaf volume [26]. However, the incorporation of protein had a positive impact, improving bread loaf volume by about 3.9–10.8%. FDH5% enhanced the specific volume of gluten-free rice bread the most (2.22 ± 0.03 cm^3^/g) (*p* < 0.05), followed by other PHs (2.12 ± 0.06 to 2.15 ± 0.04 cm^3^/g) and non-hydrolyzed proteins (2.06 ± 0.02 to 2.07 ± 0.06 cm^3^/g). This can be directly attributed to the functional properties of each protein because the amounts of other ingredients added were constant. According to the best foaming properties of FDH5%, the formation of foam during mixing as well as gas cell expansion and foam stabilization during proofing may support an increase of bread crumb dimension (Figure 5d). Moreover, protein unfolding and interactions of proteins with each other or with other ingredients may occur during baking, leading to enhanced structural properties.

#### 3.5.2. Crumb Firmness and Relative Elasticity

The effect of protein addition on crumb firmness and relative elasticity of breads is presented in Table 3. It was clearly seen that different levels of protein enrichment influenced crumb firmness differently. The least firm crumb was determined for bread with 0% protein addition (*p* < 0.05). This may be the result of a non-homogenous crumb structure containing large gas cells in the control bread, as shown in Figure 5a. The recipe with egg albumen provided the firmest crumb (65.58 ± 0.73 N), followed by that containing peanut protein concentrates (50.41 ± 0.95 N) (*p* < 0.05). The firmest crumb can be attributed to the high water-binding and gelling properties of egg albumen that can generate a homogenous bread crumb with very small gas cells (Figure 5b), resulting in greater firmness, but increased relative elasticity on the other hand. This result is consistent with the study of Han et al. [2], in which the addition of egg white solid increased the elasticity of gluten-free batters.

Among PH-containing breads, PHs with DH5% had a greater impact on crumb firmness than PHs with DH10% (Figure 5D–G). This may be due to the better foaming and emulsifying properties of partially hydrolyzed protein that can deliver a homogenous crumb structure, resulting in improved textural properties. This result had a very high correlation with relative elasticity, with a Pearson’s correlation coefficient of 0.774 (data not shown). Moreover, a moderate correlation (*r* = −0.662) between crumb firmness and specific volume was observed. A less firm crumb was obtained in bread with a higher specific volume. This result is in accordance with the studies of Han et al. [2] and Masure et al. [27], who reported that crumb firmness is largely related to bread specific volume.

## 4. Conclusions

The chemical and functional properties of proteins can be favorably modified by an effective enzyme. Partial hydrolysis can extend the functional properties of protein, especially foaming ability. The bioactivity of protein can also be enhanced by a high DH. For protein-enriched gluten-free bread formulations, the incorporation of peanut PHs with a small DH successfully improved bread qualities, such as increasing specific loaf volume and bread crumb relative elasticity. These results indicate that enzymatic technology can be used to modify some wanted properties of protein and could be useful for the development of a novel food ingredient from protein-based material.

## Figures and Tables

**Table 1:** Proximate composition of defatted peanuts and yield of protein concentrates.

	KAC431	KAC1
**Defatted Peanuts**		
Protein (g/100 g)	42.39 ± 0.18 ^a^	35.16 ± 1.15 ^b^
Carbohydrate (g/100 g)	29.42 ± 0.11 ^a^	27.03 ± 0.96 ^b^
Oil (g/100 g)	15.01 ± 1.22 ^b^	18.77 ± 0.51 ^a^
Crude fiber (g/100 g)	5.31 ± 0.82 ^b^	10.83 ± 1.52 ^a^
Ash (g/100 g)	4.04 ± 0.03 ^a^	3.46 ± 0.07 ^b^
Moisture (g/100 g)	3.83 ± 0.10 ^a^	4.75 ± 0.01 ^b^
**Protein Concentrate**		
Extraction yield (%)	43.26 ± 1.78 ^a^	37.25 ± 2.39 ^b^
Protein content (g/100 g)	77.76 ± 0.31 ^b^	74.14 ± 0.22 ^a^

Different letters in each row represent significant different mean values (*p* < 0.05). Number of replicates: *n* = 3.

**Table 2:** Amino acid profiles of peanut protein concentrates (mg/100 g).

Amino Acids	KAC431	KAC1	Amino Acids	KAC431	KAC1
**Hydrophobic**			**Electrical Charged**		
Valine	5007 ± 144 ^a^	4366 ± 38 ^b^	Histidine	1736 ± 0.00 ^a^	1621 ± 0.00 ^b^
Isoleucine	4244 ± 157 ^a^	3816 ± 231 ^b^	Lysine	2559 ± 0.00 ^b^	4493 ± 0.00 ^a^
Leucine	7885 ± 55 ^a^	7130 ± 144 ^b^	Aspartic acid	9267 ± 464 ^a^	8336 ± 373 ^b^
Methionine	777 ± 25 ^a^	817 ± 87 ^a^	Glutamic acid	21,108 ± 0.00 ^b^	21,310 ± 0.00 ^a^
Phenylalanine	6554 ± 0.00 ^a^	5883 ± 0.00 ^b^	**Polar Uncharged**		
Tyrosine	4437 ± 0.00 ^a^	3670 ± 0.00 ^b^	Serine	2638 ± 16 ^a^	2424 ± 57 ^b^
Tryptophan	1838 ± 0.00 ^a^	1732 ± 0.00 ^b^	Threonine	1426 ± 39 ^a^	1229 ± 99 ^b^
Alanine	3325 ± 0.00 ^a^	3051 ± 0.00 ^b^	**Others**		
**Special Cases**			Hydroxylysine	<20	<20
Proline	4594 ± 151 ^a^	4119 ± 168 ^b^	Hydroxyproline	69 ± 3 ^b^	80 ± 6 ^a^
Glycine	3893 ± 0.00 ^b^	4072 ± 0.00 ^a^	**Essential Amino Acid**	30,290	29,466
Cystine	846 ± 0.00 ^b^	3467 ± 0.00 ^a^	**Total Amino Acid**	82,223	81,636

Different letters in each row represent significant different mean values (*p* < 0.05). Number of replicates: *n* = 2.

**Table 3:** Quality parameters of gluten-free breads.

Recipes	Weight Loss (%)	Specific Volume (cm^3^/g)	Crumb Firmness (N)	Relative Elasticity (%)
Control	12.56 ± 0.12 ^b^	1.98 ± 0.01 ^d^	40.16 ± 1.82 ^e^	52.88 ± 0.33 ^e^
Egg albumen	12.72 ± 0.23 ^b^	2.07 ± 0.02 ^c^	65.58 ± 0.73 ^a^	63.56 ± 1.37 ^a^
Protein concentrate	12.57 ± 0.26 ^b^	2.06 ± 0.02 ^c^	50.41 ± 0.95 ^b^	54.66 ± 1.22 ^d^
ADH5%	13.41 ± 0.24 ^a^	2.14 ± 0.05 ^b,c^	47.46 ± 1.03 ^c^	56.18 ± 0.20 ^c^
ADH10%	13.59 ± 0.29 ^a^	2.15 ± 0.04 ^b^	48.35 ± 1.28 ^b,c^	58.69 ± 0.54 ^b^
FDH5%	13.34 ± 0.18 ^a^	2.22 ± 0.02 ^a^	43.65 ± 1.38 ^d^	57.98 ± 0.66 ^b^
FDH10%	13.37 ± 0.29 ^a^	2.12 ± 0.06 ^b,c^	45.71 ± 2.84 ^c,d^	59.11 ± 0.98 ^b^

Different letters in each row represent significant different mean values (*p* < 0.05). Control: 0% protein added, whereas other recipes were constantly added at 2% protein. Number of replicates: *n* = 8.
